# Developmental Assessment of Visual Communication Skills in Primary Education

**DOI:** 10.3390/jintelligence10030045

**Published:** 2022-07-22

**Authors:** Tünde Simon, Ildikó Biró, Andrea Kárpáti

**Affiliations:** 1 Department of Pedagogy, Hungarian University of Fine Arts, 1062 Budapest, Hungary; simon.tunde@mke.hu; 2 Doctoral School of Education, University of Szeged, 6720 Szeged, Hungary; biro.ildiko@edu.u-szeged.hu; 3 Department of Communication and Media Studies, Corvinus University of Budapest, 1093 Budapest, Hungary

**Keywords:** visual communication, visual skills and abilities, developmental assessment, digital assessment tools

## Abstract

In this paper, we describe subskills of visual communication based on the skill structure outlined in the Common European Framework of Visual Literacy. We have developed this Framework further through assessing the development of subskills related to visual communication in the “produce” and “respond” domains of CEFR-VC in primary school grades. We developed and validated online digital assessment tools to facilitate the introduction of authentic assessment as a standard practice in curriculum development. The results of this study include the definition of its components, development of innovative tools for their assessment, and description of the development of its subskills in the “produce” and “respond” domains. Our tests for the “respond” domain of the visual literacy framework were administered in the eDia interactive diagnostic testing environment in Grades 4–6 (ages 10–12 years) of the Hungarian primary school system. The tools for the second experiment about the “create” domain of visual communication were developed in the GeoGebra free educational software environment and tested major components of the “produce” domain of visual communication in primary Grades 5–8 (ages 11–14 years). Results show increasing attainment in subskills through the age groups in the “produce” domain and less significant or no development in the “respond” domain, which is underrepresented in Hungarian art education curricula. Development is unrelated to school achievement in non-art disciplines, showing the distinctiveness of the visual domain, and is weakly related to gender and digital literacy. Using our subskill descriptions and the assessment tools, teachers may select those subskills that they find most important to develop during the limited teaching time for visual arts. The paper ends with suggestions to enhance visual communication as a cross-curricular competency that develops visual-spatial intelligence.

## 1. Introduction

*Spatial intelligence*, the capacity to construct mental representations of objects and people in space and perform activities based on spatial perception, to evaluate and modify those perceptions according to subsequent experience and recreate components of a perception even without the physical presence of the original stimulus, is one of seven intelligences described in the theory of multiple intelligences (MI). *Visual skills* are mentioned here as a modality central to spatial intelligence that also incorporates other senses and cognitive operations. The creation of objects and images (from artworks to graphs) was indicated but not detailed, as Gardner mentioned artists, architects, engineers, surgeons, and sportspeople who require high degrees of spatial intelligence to act successfully. (Gardner 1983, pp. 173–75).

In his reinterpretation of the theory of intelligences, Gardner discussed *visual intelligence*, which utilizes perception and manipulation guided by sight; and *spatial intelligence*, which involves creation and perception of space and does not necessarily require vision (as in the tactile exploration of persons with damaged eyesight). In the expanded MI model, *visual-spatial intelligence* is activated during the creation, perception, interpretation, and use of spatial and two-dimensional representations. Gardner offers a broad definition of intelligence, involving high-level and ordinary applications: “An intelligence entails the ability to solve problems or fashion products that are of consequence in a particular cultural setting and community. The problem-solving skills allows us to approach a situation in which a goal is to be obtained and locate the appropriate route to that goal. The creation of cultural products allows one to capture and transmit knowledge or to express one’s conclusions, beliefs, or feelings. The problems to be solved range from creating an end for a story to anticipating a mating move in chess to repairing a quilt”. (Gardner 2006, p. 12). The accurate perception of objects, spaces, people and images and their relations, and the construction of mental representations of visual information involves interrelated spatial and visual skills. 

In related studies, visual-spatial intelligence is always mentioned in conjunction with acts of perception and creation. The creative subskills connected to visual-spatial intelligence were first reduced to representation, then creative use of visual language (e.g., symbolization and expression of moods, feelings, and thoughts) were identified as important manifestations of this intelligence (Winner et al. 2013). Gardner’s definition of visual-spatial intelligence inspired studies to describe its components and prove its importance for other areas of learning. (An example: Stavridou and Kakana 2008) compared the use of three graphic abilities related to visual-spatial intelligence with performance in science and mathematics and found significant correlations). In his phenomenologist critique of the interpretation of visual intelligence in the theory of MI, Cary (2004) called for an enrichment of the concept with considerations of the use of visual language for aesthetic expression and called for research on its genesis and evolution. Development of visual-spatial intelligence was one of the main objectives of Gardner’s work at Harward Project Zero (n.d.) (The research group was founded by Nelson Goodman at the Harvard Graduate School of Education in 1967 to explore learning in and through the arts). The “zero” in its name suggested that no previous studies were considered relevant in this area as they provided emphatic claims for more arts at schools, but little research evidence. Visual art education projects that Gardner and his associates initiated focused on the varied and flexible use of visual language, and represented a radical shift from contemporary, high-arts-based methodologies (e.g., Hetland et al. 2007). Visual-spatial intelligence was explained through educational interventions, but levels of the development of skills that constitute visual-spatial intelligence were not described.

In the 1990s, more and more studies on the development of certain visual skills were published and the need for a common framework to guide research for curriculum design was voiced. (Boughton 2013; Winner et al. 2013). Art education worldwide experienced a “*cognitive turn*” and wanted to show its potentials (Rat für Kulturelle Bildung 2019). In response to the need for a research-based structure of knowledge, skills, and competencies constituting the use of visual language, art educators from 17 European countries established the *European Network of Visual Literacy* (ENViL n.d., https://envil.eu/, accessed on 4 June 2022). The consortium analyzed 27 European art and design education curricula to identify skills that these educational documents found important to teach in public education (Kirchner et al. 2016). The prototype of the *Common European Framework of Reference for Visual Literacy*, (CEFR-VL) was developed. In compliance with other frameworks defining various important literacies in the field of native language use or mathematics, the framework was named “literacy”, although dealing with images (not letters) and their use. The competency definition of Franz Weinert (2001) was employed, who claims that a competence requires the combined use of learnable knowledge, skills, and attitudes. It is demonstrated in specific (professional) situations; its outcome may be an object as well as a demonstrable behavior or disposition.

**Figure 1:**
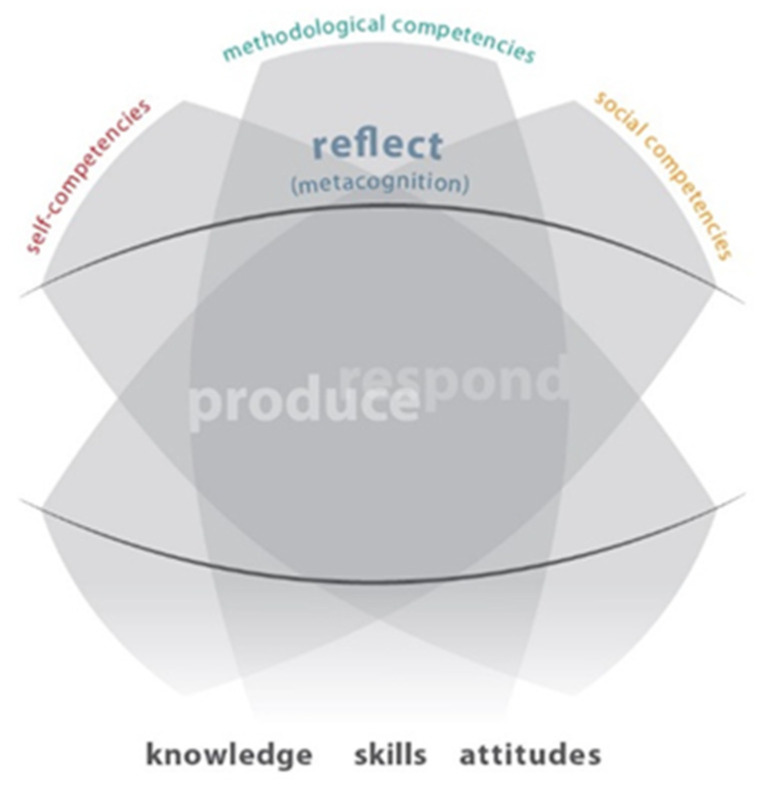
Structure of the Common European Framework of Visual Literacy (CRFR_VC (ENViL Structural Model 2016)).

**Figure 2:**
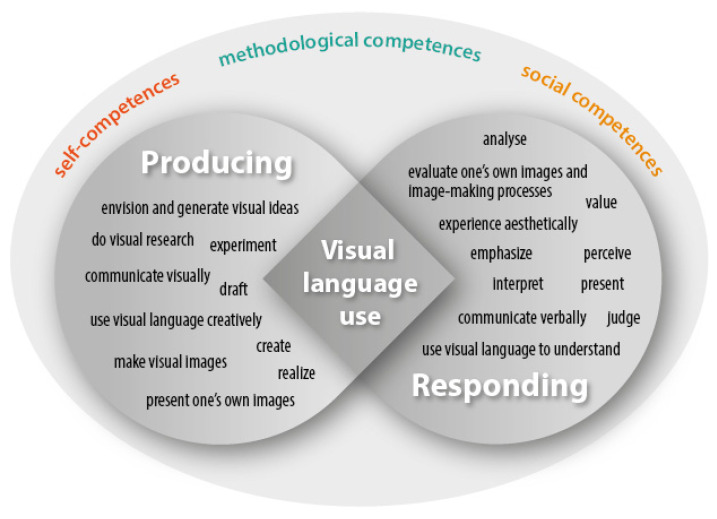
Skills and subskills of the Common European Framework of Visual Competency (CRFR_VC) by Ildikó Biró, 2022, based on (Schönau et al. 2020).

A series of (inter)national experiments to test its validity and usability for curriculum design and research on skills development were launched and published (Wagner and Schönau 2016). While the concept of visual-spatial intelligence was defined as a construct that needed verification, ENViL offered a new approach to visual-spatial intelligence through the identification and description of skills and subskills identified in educational curricula that defined teaching practice. The Framework was revised to include more subskills and renamed to the *Common European Framework of Reference for Visual Competency*, (CEFR-VC) (Schönau et al., Figure 1 and Figure 2).

The framework identifies two major competence domains (skill clusters): *producing* (planning and realization of a visual creation) and *responding* (perception and response to a visual creation), while acknowledging their interrelated character in certain visual activities. Relationships of visual skills with basic competencies: *self-, methodological, and social competences*, are also indicated. Metacognition represented above the two domains or skill clusters indicate the importance of critical awareness of the thinking and learning processes of our own and those of others. Metacognition as part of visual competence indicates the importance of monitoring and assessing our creative and responsive performance. 

Figure 2 represents the subskills identified in the revised version of the framework (Schönau et al. 2020). They are grouped under the domain they belong to. However, some subskills may play a role in activities requiring the other domain as well, depending on the situations they are employed (Billmayer 2016). They are defined extensively in previous publications (Wagner and Schönau 2016, pp. 66–79; Schönau et al. 2020). For eleven subskills, there was enough research evidence to describe three levels of attainment (elementary, intermediate, and competent, cf. Wagner and Schönau 2016, pp. 80–90). Further research is needed to establish age-based performances that may better serve the goals of curriculum innovation as these studies better support the definition of output requirements for school grades. This study is one of these efforts. 

Our investigations of the development of visual skills and abilities are based on the CEFR-VC framework. Our intention is to define age-based development through assessment tools that are authentic both as research instruments and art tasks. Our assessment efforts intend to support educational interventions; therefore, we do not define three levels of attainment, as in the CEFR-VC, but describe the performance of students at different school grades and thus facilitate curriculum development and assessment that is responsive to age-related performance. In this paper, we present the assessment of subskills of the two major domains (skill clusters) of visual communication. Our instruments were designed for digital testing, to ensure easy access by art educators who can administer them in the school computer laboratory without the need of printing test sheets in color. The tasks are organized in tests but may also be used individually for practice or assessment. 

## 2. Materials and Methods

### 2.1. Assessing Subskills of the Produce Domain of Visual Communication

Our first experiment presented in this paper focuses on the assessment of the “produce” domain of visual communication. Our online tool includes tasks that can be used in everyday pedagogical practice, for students in Grades 5–8 of the Hungarian primary school system (ISCED 2). Previous studies (Kárpáti and Gaul 2011; Schönau 2012; Frick 2018) have shown that the combined assessment of skills of visual creation and perception of/response to visual stimuli is difficult and can be limited to a few subskills. Therefore, our assessment tool focuses on creative tasks only, as outlined in the “produce” domain of the CEFR-VC. In designing the tasks, we aimed to include all possible visual creative skill elements of visual communication. If we want to develop authentic tasks, it was impossible to represent a single activity in isolation, and therefore multiple skill elements are represented in each task. The test tasks are the first step in developing a digital task bank for the assessment of visual communication.

The test aims to assess four subskills of visual communication—composition, abstraction, creation of visual symbols, and *modality shift*—selected from the 19 identified and validated skill elements of the Hungarian Visual Framework (Kárpáti and Gaul 2013). and the CEFR-VC. (Table 1).

Skills of the “produce” domain that are not integral parts of visual communication and those that are not suitable for online assessment (e.g., the use of artmaking tools) have been excluded from the test. It contains only a limited number of tasks about spatial composition (a sophisticated set of visual skills that can only be measured reliably through a complex set of tasks, cf. Kárpáti and Babály 2021). The tool developed for the “produce” domain does not assess the visualization of sound, as this example of modality shift contains subjective interpretations that is captured and evaluated through qualitative measures in the second experiment reported here. The current study of the creative subskills of visual communication involved composition in 2D, creation of visual symbols, abstraction, and modality shift described in Table 1. Our intention was to create a digital assessment tool that can be employed to evaluate these important components of visual literacy and reveal correlations among them.

#### Assessing Subskills of the “Respond” Domain in Visual Communication

The subskills related to visual communication are only a small part of the “respond” domain. When we perceive images, people, objects, or spaces, we combine past experiences and knowledge with a current visual sensation. The “respond” domain spans the visible world from everyday objects to works of art, from intimate to cosmic spaces. When designing an assessment tool for these subskills of visual communication, we focused on those that are most relevant for everyday life and the world of work. These subskills are also developed at school, so their assessment is relevant for art educators who need feedback for improving their teaching and learning programs. We designed tasks that involve *visual recognition and differentiation* among forms: *visual interpretation*—connecting a text with the related image, connecting messages of a literary work with its illustration; *visual analysis*—reading graphs and charts, interpreting dynamic visualizations, interpreting color symbols and spatial relations. 

We developed tasks with 119 assessment items, the solvability of which is not affected by disciplinary knowledge. We sought to choose topics that were equally motivating for both sexes. The images of the tests resemble those used in everyday life: pictograms and other types of symbols, illustrations from storybooks, works of art, photos, and children’s drawings.

The subskills we assessed were *visual recognition, visual interpretation, and visual analysis.* Descriptions of the skills and task types are summarized in Table 2. In the “respond” domain, we selected skills from the CEFR-VC framework and defined their subskills. In the assessment project, we showed how different age groups perform in the tasks related to the levels.

### 2.2. Sample and Assessment Methods of the Two Studies

#### 2.2.1. Sample and Task System for the “Produce” Domain of Visual Communication

312 students from 4 schools completed the test (Grade 5, age 11 years: N = 77, Grade 6, age 12 years, N = 78, Grade 7, age 13 years, N = 76, Grade 8, age 14 years, N = 81). None of the students attend a class with special art instruction. 

Test items were developed with the use of the Notes function of the GeoGebra software. The test included seven tasks in a sequence to represent varying levels of difficulty of the use of the testing software. The digital test contains seven complex tasks with 23 items. In designing the tasks and developing the scoring criteria, we aimed to focus the tasks on four subskills of visual communication: 2D composition, abstraction, symbolization, and modality shift (see Table 1 for a description of the subskills). 

The use of the GeoGebra toolset was introduced to the students through video tutorials demonstrating the use of tools needed for each task. The tutorials could be re-watched, and it was also possible (but not compulsory) to experiment with the use of the tools on practice tasks. The tasks were included in the test according to the complexity of the tool use, with easier items first. Some of them are described below.

##### Highlighting a Shape Using Color Contrasts

This task involves the application of the fundamental categories of color contrast (hue, saturation, light/dark, cold/warm, complementary, analogous, saturation, and extension). The decorative and attention-capturing effect of colors must be consciously applied. Two groups of houses have to be colored, using different contrasts, so that the house in the middle becomes the focus of attention. Evaluation of solutions was based on the success of highlighting with color.

##### Creation of Graphic Symbols

**Figure 3:**
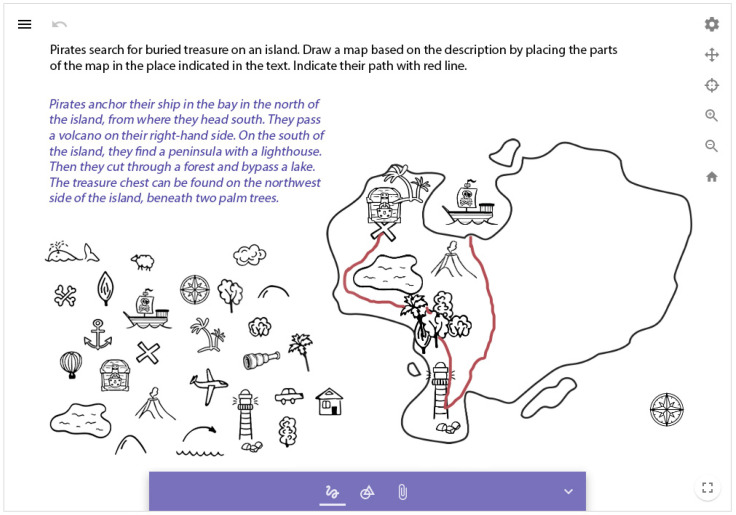
Creating a map with graphic symbols.

The task is to create a pirate map. Based on the text that explains the landmarks to be represented on the map and their placement, students have to represent semantically, visually, and structurally important parts of the map. Then, the route of the pirates should be represented (Figure 3). 

Since GeoGebra offers limited possibilities for symbol creation and customization, the visual symbols typically found in paper-based tests were employed in the digital version.

##### Figurative Composition Created from Geometric Shapes

The task works on the principle of a tangram puzzle, an image-assembly task that focuses on the rearrangement of seven separate pieces into a complete image of various shapes (in outline or silhouette only). Tangrams help to understand visual-spatial relationships and develop the skill of mental rotation (Siew et al. 2013). Compared to traditional toys, a virtual tangram requires the development of a mental plan for the correct rotation of the pieces. A trial-and-error problem-solving strategy cannot be applied. In our task, the students have to assemble a fish and a bird from 7 elements. In GeoGebra Notes, it is not possible to avoid the overlapping of elements, nor can they be fixed in size. This fact significantly influenced the solutions.

**Figure 4:**
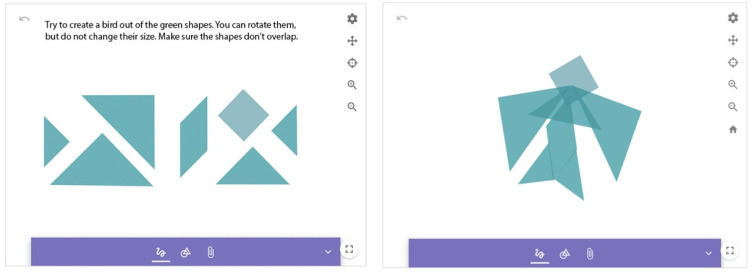
Figurative composition 2.

The evaluation criteria involved the use of all tangram elements to build the required image; the overlap between elements; change of element size; success in creating a bird shape and a fish shape (Figure 4).

##### Expressive Value of Geometric Shapes

**Figure 5:**
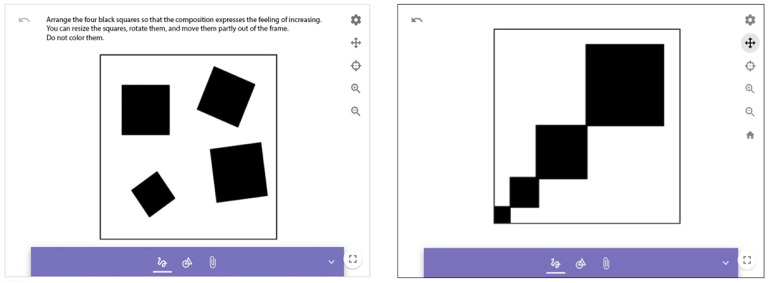
Geometric shapes.

This task is the adaptation of the so-called *black square problem*, which was proven to be an effective developmental tool for visual composition (Wilde and Wilde 2000). Understanding and applying its operation and basic design rules help to convey a message effectively in visual communication. To create a successful solution, students have to create a geometric shape using certain prescribed criteria. These include cropping, framing, overlapping, space and the illusion of space, size, direction, position, perspective, and positive and negative relationships. With the use of four black squares, students have to create compositions that represent two concepts: *order* and *increase* (Figure 5). Here it is important to watch the optional video tutorial provided for all tasks. Assessment criteria involve the placement of elements for expressiveness: size, arrangement, axis, and alignment. Such tasks are highly adaptable to the digital environment and may be used for developmental assessment in visual arts (Schönau 2012) to discuss concepts through visualizations.

##### Two-Dimensional Spatial Representation

**Figure 6:**
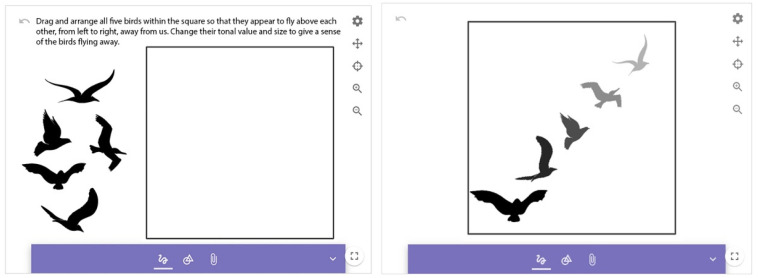
Two-dimensional spatial representation.

The representation of space in two dimensions is the way in which depth, the difference in size, and distance are represented using 2D pictorial elements. In this task, bird-like shapes must be arranged, a spatial illusion has to be created through size difference, and tonal gradation has to be used to indicate the direction of the flight of birds from left to right, moving away from the spectator (Figure 6). This requires selecting, resizing, and rotating visual elements as well as changing their tonal value. The assessment criteria are (1) toning, (2) scaling, (3) composition indicating depth, and (4) direction.

##### Visual Rhythm

Although the task may seem simple, it involves the use of several basic components of visual communication. This task assesses developing visual rhythm using form and shade. This task did not produce a wide variety of solutions, due to the limitations of the online interface.

#### 2.2.2. Sample and Task System of the Response Domain of Visual Communication

In most tasks, we needed to develop multiple items to avoid blind guessing and increase reliability. Some tasks were innovative, such as the sound and image modality change. In Table 3 we summarize the content of the tasks: image types employed, and the number of tasks and items designed for the assessment of the subskill.

Task sets of the three subskills included in our tests will be presented through the description of one task from each set. All tasks were realized in eDia, the online, interactive, diagnostic assessment environment (Molnár and Csapó 2019).

##### Recognition and Differentiation

**Figure 7:**
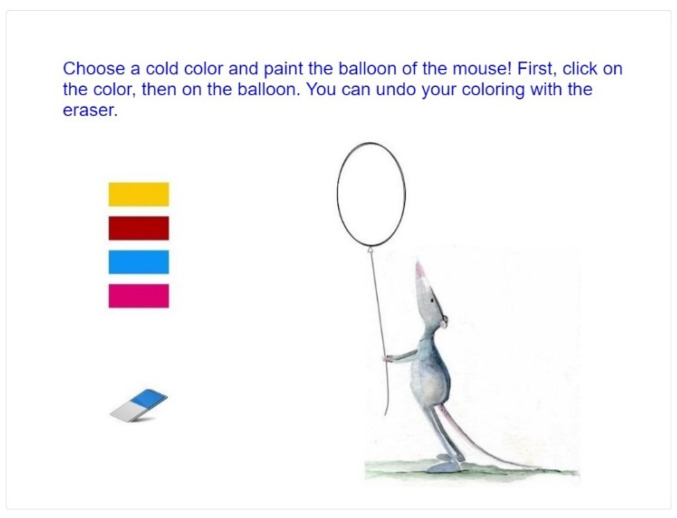
Recognition and differentiation task (task for Grade 5, age 11 years).

For the subskill of recognition and differentiation, we developed 40 tasks. Recognition of form and color, knowledge of color contrasts, and part-to-whole matching are needed for the realization of the task presented on Figure 7. 

##### Visual Interpretation and Recognition of Connections

**Figure 8:**
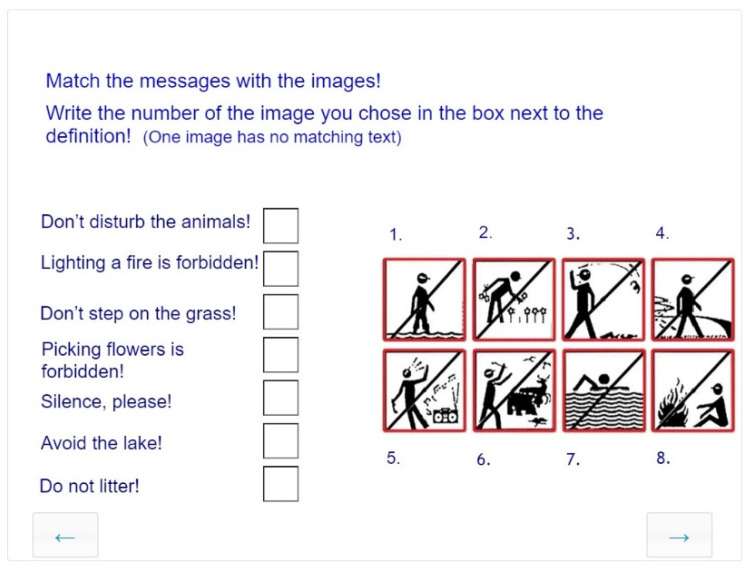
Task for interpreting symbols and recognition of visual connections for Grade 6, age 12 years.

The symbolization task on Figure 8 relates to the subskill of visual interpretation that involves attaching meaning to abstract images. A frequent visual communication problem to be solved is the interpretation and recognition of connections between traffic signs or pictograms in public spaces and their content. More sophisticated subskills of this task set involve map reading and recognition of messages conveyed in simple linear drawings that use basic elements of visual language (dots, lines, geometric shapes, patches), or works of art with a clear communicative intention (such as Fluxus or other conceptual movements in 20th century art).

##### Visual Analysis: Reading Messages Conveyed in Abstract Compositions

**Figure 9:**
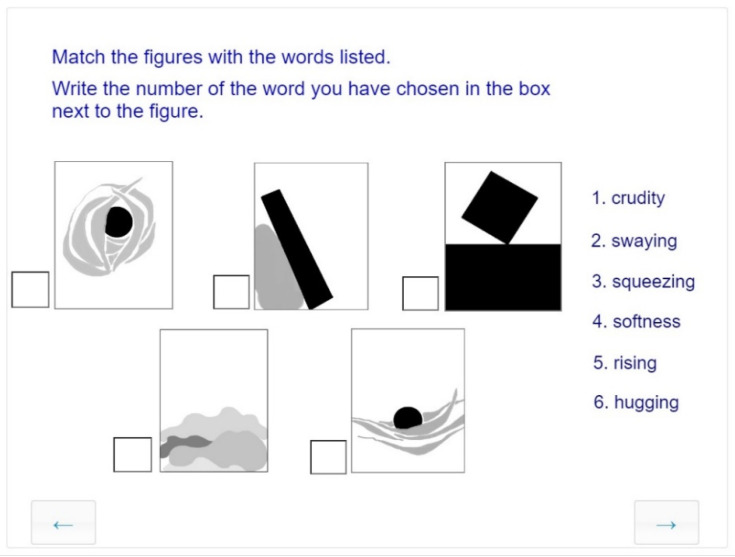
Example from a series of tasks designed to assess the abstraction subskill.

As part of visual communication, abstraction is the creation of signs and shapes by highlighting, simplifying, and reducing (Figure 9) Abstract compositions may convey meaningful signs and symbols. Carefully composed visual effects may evoke the same emotions or thoughts in different viewers. Our interpretation of visual elements is based on conventions transmitted through real-life experiences and organized through educational interventions. To communicate effectively through signs and symbols, the rules of visual perception should be acquired. In designing the test items, we used the tasks and illustrations of Paul Klee’s Pedagogical Sketchbook (Klee [1953] 1973). Paul Klee developed a collection of basic symbols (graphemes) such as dots, wavy lines, staggered lines, and open shapes that allow for multiple interpretations. We adapted 36 items to our task set for the subskill of visual analysis.

##### Modality Change

**Figure 10:**
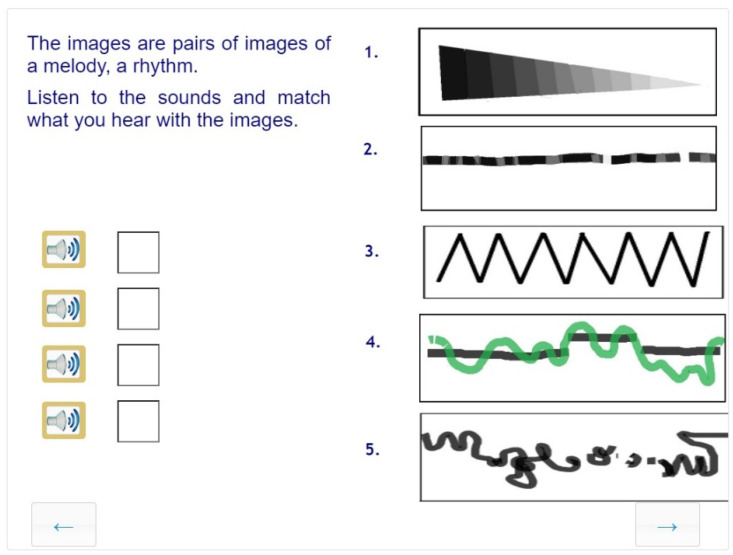
Sample task for modality shift: a task designed to interpret the visual and auditory modality. This anchor item was solved in all three grades.

Most visual tasks require some degree of modality change. The required content of an image is provided through a verbal description, or an artwork has to be analyzed through words. This task is different; here, the modality of sound is involved, and images have to be matched with a brief musical piece. Twenty-five such items were developed and piloted to see if they worked. Relying on the mood and rhythm and the tonal qualities (raising and lowering of pitch and dynamism of presentation), many students were able to find the equivalents of the musical pieces in visual images. (Figure 10). 

## 3. Results and Discussion

The tests were taken in school settings, in the computer laboratories on laptop computers or in a classroom on tablets. We calculated the reliability indicators for both tests and used Cronbach’s alpha to reveal internal consistency. We employed an independent *t*-test to measure differences between genders and students using different digital devices. One-way analysis of variance (ANOVA) was used to determine whether there are any statistically significant differences between age groups and whether school results in different disciplines, or if the frequency of playing computer games influences performance in visual communication tests. As the CEFR-VC framework lists major skills only, our hypothesized structure of the visual communication subskills in the “produce” and “respond” domains were verified through confirmatory factor analysis. The results are summarized below.

In the pilots, we tested the relevance of the platform for results and did not find correlations (cf. Table 4 below). The medium made no difference; paper-based test scores and digital test scores showed similar statistical values. Taking the tests on laptops and tablets and receiving results immediately facilitates the work of art educators who may use our tools for continuous, formative assessment as well as end-of-term, summative testing of skills. This finding is also important because of the increased relevance of digital imaging in private life and in the workplace. Paper-based tests are becoming less and less authentic as more and more programs in visual art education involve digital media. (Table 4). 

### 3.1. Assessment Results: Subskills of Visual Communication Related to the Produce Domain of CEFR-VC

All tests for both domains underwent validity and reliability analysis. The reliability of the whole test is good (Cronbach’s alpha > 0.8). The high reliability is due to the high variance and the resulting heterogeneous group composition (Table 5).

To reveal differences in performance of the age (and grade) of students, we performed a one-way analysis of variance (ANOVA). There was a measurable difference between the age groups.; this was confirmed by the high partial Eta-squared effect size value (η_p_^2^ = 0.30). The significance level of the Levene’s test was 0.03 (*p* < 0.05); we used the Dunnet T3 test to reveal age-related results. Two groups were separated: 11–12 and 13–14-year-olds (Table 6).

The test results presented in Table 7 show no relevant difference between the scores of the boys and girls, in contrast to previous studies performed with paper-based tests (McGivern et al. 1997; Abramov et al. 2012; Siu et al. 2015). While in paper-based tests, manual dexterity is an important and often dominant subskill in digital imaging, a wide variety of other subskills are involved. 

Task-level analyses show that there is not much difference between the mean and variance distribution of each task. The average task solution rate ranges between 41% and 61%, which is considered acceptable. The standard deviation is between 26% and 45%, indicating that some tasks are too easy, or their assessment criteria are problematic (Table 8). These tasks will be corrected in the published assessment tool.

The elimination of the gender performance gap using these digital toolset results is partly due to the absence of freehand drawing among the tasks, as emphasized above. Another factor often mentioned as affecting performance may be the different digital literacy level of the two genders, especially the more intensive online gaming habits of boys. Girls play computer games less frequently, and therefore may experience difficulties in using the functionalities of a complex online test. (Veltri et al. 2014). In our case, no such difficulties were experienced. The type of digital instrument used produced no significant differences in the results of students, either. One part of the students took the test on personal computers (N = 104) and the other part took it on tablets (N = 182). There is no correlation (r = 0.07) or significant difference between test scores and the device used (Table 9). However, the small effect size suggests that it would be advisable to repeat the measurement on a larger sample.

**Figure 11:**
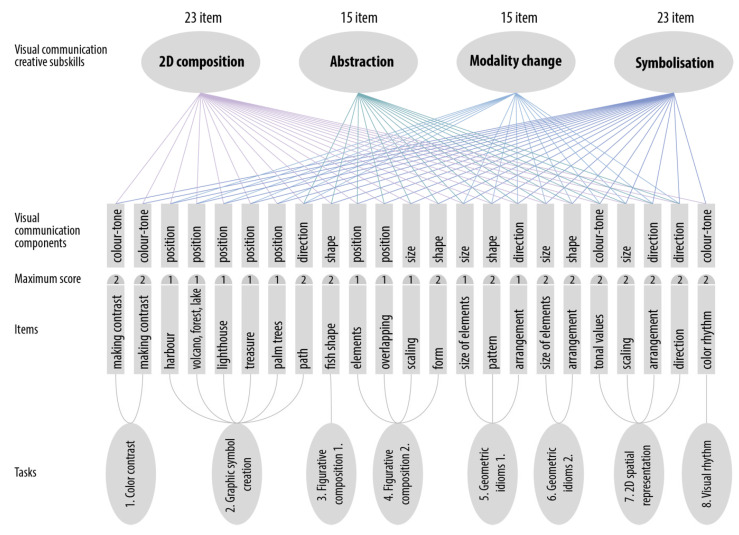
Digital test of visual communication: “produce” domain, Subskill structure of the tasks.

In designing the digital test and developing the scoring criteria, we aimed to focus the tasks on the four subskills of visual communication described in Table 1: 2D composition, abstraction, symbolization, modality shift. We considered isolating and assessing these subskills, focusing only on one of them in each task. However, when the preliminary structure was developed, we realized that the subskills we examined are likely to be activated together. It seemed to be impossible to represent only one subskill within a single task. More than one subskill that we intended to assess was activated during the solution of a visual communication task. Based on the assessment results, it was confirmed that the four creative subskills of visual communication that we focused on are closely interrelated (Figure 11).

A solution to this problem was to narrow the analysis down to a single skill element, the use of compositional principles and components in *two dimensions*. Our attempt to separate the compositional components by item is shown in Figure 11, in the row labeled “visual communication component”.

According to the new approach, five visual subskills were separated at test-item level (creation of color-tone, position, direction, shape, and size). Our hypothesis was confirmed by a factor analysis reduced to five factors, which showed a 70% match with the components of visual communication skills based on the literature. There was a weak-to-medium correlation between the separated visual communication components. The strongest correlation was between direction and shape (r = 0.57) and direction and position (r = 0.57); the weakest correlation was between shape and position (r = 0.30). However, all the visual communication components were highly correlated with the overall test, i.e., they showed a strong relationship with the overall test score. The co-occurrence of subtest behaviors and the strength of the relationships between the visual communication components support our hypothesis that the test assessed related but not identical visual communication components.

In further development of the test, it would be worthwhile to concentrate on these components. An attempt to assess the use of additional components and principles of composition (balance, emphasis, movement, contrast, pattern, rhythm, uniformity/variety) would be desirable, as these together form the basis of the language of visual communication.

### 3.2. Assessment Results: Subskills of Visual Communication Related to the “Respond” Domain of CEFR-VC

Our research sample consisted of students of Grade 4, 5, and 6 of the compulsory Hungarian primary school system. No classes with special art instruction were included. A total of 21 classes from 13 schools completed the Grade 4 test, with an average age of 10.6 years. The Grade 5 sample consisted of 338 students from 26 classes in 14 schools, with an average age of 11.4 years. Year 6 was represented by 486 students from 26 classes in 16 schools. The average age of the 6th-graders was 12.5 years. A total of 1256 students participated in the assessment project.

A total of 24 items were developed for the tests of the three grades. The reliability of the test interpreted by the anchor items was good (Cronbach’s α = 0.78). Table 10 summarizes the psychometric characteristics of the tests for the three grades.

The test was also examined through probability test theory. The EAP/PV reliability index is appropriate for all Grades (Table 11). The analysis showed that the items cover the average skill level, shifting slightly to higher values. The test has a perceptible ceiling effect, so it separates learners who achieve higher results less than expected.

A confirmatory factor analysis (CFA) was performed a to verify the hypothetical structure of the subskills (Table 12). The comparative fit index (CFI) indicates the model fit by examining the discrepancy between the data and our hypothesized model; the Tucker–Lewis Index (TLI), also known as the non-normed fit index, was used for linear mean and covariance structure modeling. Root-mean-square error of approximation (RMSEA) was used to measure of the estimated discrepancy between the population and model-implied population covariance matrices per degree of freedom. We realized that our model about major components of the “respond” domain of visual communication skills is weak because the subskills are multidimensional, and there is a significant correlation among the items. 

We examined how the differences between the subsamples of grades were reflected in output (Table 13). In the analysis of variance, no significant differences were found between the grades (Levene’s test = 0.35 *p* = 0.7; F = 0.11 *p* = 0.89), but small improvements were detected. 

When we examine the sample according to the year of birth (which is a more accurate age definition than school grade), the analysis of variance shows more pronounced differences according to results of Levene’s test, an inferential statistic used to assess the equality of variances for a variable calculated for two or more groups. (3.63 *p* < 0.05; F = 4.1 *p* < 0.05). While we detected a steady increase in performance in the “produce” domain, in the “response” domain we found no significant difference between the development of the age groups. We can only detect slight improvement between those born in 2001 and 2002 (t = −3.88 *p* < 0.001). The reason for this lack of development is related to the Hungarian art education curriculum, emphasizing production over reception and interpretation of images.

As in the “produce” domain, we found no differences between the performance of boys and girls (r = 0.17 for boys and r = 0.41 for girls). However, in the “response” domain, we found correlations of medium strength among learning results and test performance in the three grades (r = 0.48; r = 0.46; r = 0.46). This result calls attention to the findings of Stavridou and Kakana (2008), who found correlations between the level of certain visual skills and performance in mathematics: in the “response” domain, we also found that visual communication skills are better developed with successful learners. In the “produce” domain, however, no correlation with learning results was detected. 

## 4. Conclusions

In this paper, we introduced two assessment projects that target the competence structure of visual communication, arguably the most important visual skill cluster of our time. The first project focused on activities related to the perception of images and assessed their development through online, interactive tests administered in the eDia online, interactive testing environment in the Hungarian compulsory primary school system Grades 4–6 (ages 10–12 years). The second experiment was performed through the GeoGebra software and tested major components of the creative domain of visual communication in Grades 5–8 of the Hungarian primary school system (ages 11–14 years). Our results show increasing attainment in subskills through the age groups in the “produce” domain and less significant or no development in the “respond” domain. These findings are in line with the analysis of our art education curriculum, which is creation-focused. Such an approach does not adequately develop perception, analysis and interpretation of art works that constitute the subskills of the "Response” domain.. An important educational-practice-oriented finding of our research is that visual communication, a key life skill of the visual competency cluster, needs more intensive educational interventions in the “response” domain.

We could not identify any other standardized digital assessment tools for students of compulsory primary education (ISCED 2) in visual art education; our most significant result in this area is proving that authentic assessment of visual skills may be performed through digital tests simulating authentic use of predominantly digital visual language of children and youth. Our test items can or should never replace genres and techniques traditionally employed for detecting visual talent or assessing art performance since the foundation of the first art academies in the 1980s but may provide an alternative if skills such as visual communication are assessed that are practiced mainly through digital media. 

In comparison to paper-based tasks, where girls often outperform boys from preadolescence, we found no differences between genders. Creating digital images seems to be a skill set that is equally present in both genders of Generation Alpha, (children born in the 2010s), the cohort following the much-researched Generation Z. This finding has a significant relevance for art education: digital imaging is a medium that can be smoothly introduced in art education curricula if the software environment used produces adequate help and the tasks are age-related.

To develop art programs that are individualized and responsive to social, and cultural needs, detailed description of visual skills and subskills are needed, with data on attainable performance in age groups that can be translated to school grades. In a vocational secondary school, different subskills are needed for each group of professions and their acquisition is key for successful actions at the workplace. Our toolsets assist curriculum development as it significantly facilitates the description and mapping of the “produce” and “respond” domains of visual communication. Art education programs using these valid, reliable, and coherent instruments may introduce more focused educational interventions. 

Teachers may use our skill descriptions and tasks as an inspiration for the development of teaching programs focusing on relevant subskills and use our tasks in a test or individually for assessing the results of their interventions. In secondary grammar schools, knowledge of visual signs and symbols or methods of scientific visualization may be a valid interdisciplinary skill set. Development of visual communication skills through art education does not seem to be enough: an overarching enhancement introducing visualization genres applicable for different school disciplines may produce development.

We intend to continue developing age-related version of our tests to be used for development and assessment. Our results suggest that the subskills of the “produce” and “respond” domain of visual communication can be revealed through assessment tasks but cannot be separated due to their interrelated employment in any given task. In both domains, several subskills are activated when a single task is completed. Therefore, it is impossible to design a single-subskill task, but we can define the subskills that play a role in a task and indicate their relative importance.

We also want to expand the age range of our study to Kindergarten age groups (3-6 years) to see how frequent encounters with digital images influence visual competence at the age of the genesis of visual language. We want to create more flexible assessment instruments to avoid the ceiling effect through dynamic adaptive testing. Some of our visualizations may be transferred to the smaller screen of the smartphones and may thus offer a play-like activity for students on an easily accessible platform. This way, art educators may include short assessment periods or game-like tasks of edutainment quality in their lessons. A very interesting venue to explore will be research on the effects of visual communication genres that transmit science knowledge (e.g., multimedia infographics, flowcharts, modifiable graphs, and simulations). Visual communication supports learning and may be especially beneficial for poor verbalizers. 

Our further research projects will also target the relationship of the skill clusters “produce” and “respond”, as represented in the Common European Framework of Visual Competency (Wagner and Schönau 2016; Schönau et al. 2020). Does high performance in the “produce” or “respond” cluster influence performance in the other? In this respect, a more sophisticated approach seems to be promising: which subskill of one cluster corresponds with the other, offering new potentials of development? 

## Figures and Tables

**Table 1:** The subskills selected to assess the “produce” domain of visual communication.

Subskills	Explanation of Concept	Activities Related to the Subskills
Two-dimensional (2D) composition	Creating and arranging visual elements. Use of colors.	Organizing and emphasizing visual elements of a pictorial representation. Creating visual dynamics, rhythm, hierarchy. Assigning meaning to certain forms through spatial arrangement. Use of color to direct attention, color contrasts, hues, tones, saturation.
Creation of visual symbols	Creating symbols, allegories, visual metaphors, and metonyms.	Making explanatory diagrams, pictograms, logos, and maps. Representing a word, concept, or description by creating a unique symbol or symbol system (and hierarchy within it).
Abstraction	Creating visual signs and shapes by highlighting and simplifying images. Creating shapes with specific meaning. Conventional, rule-based signs and images, visual representation of proportions. Expression and representation of real or imagined relationships, representing time and movement.	Visual representation of data, relationships, concepts, structures, and proportions. Creating explanatory drawings, diagrams, flow charts (e.g., map, path, assembly drawing). In several visual genres, symbolization and abstraction are both present.
Modality shift	Visualization of narratives.	Making a picture to illustrate a written or orally transmitted text.

**Table 2:** The subskills selected to assess the “respond” domain of visual communication.

Skill	Subskills	Activities to Assess Subskills
Visual recognition, differentiation	Recognition of two-dimensional (2D) forms 1	Recognition of visual signs (e.g., dots, lines, spot, tone, color, form). Recognition of the meaning of signs in familiar context.
Visual interpretation	Recognition of two-dimensional (2D) forms 2	Recognition of simple visual signs and sign groups in familiar and new context.
Visual analysis	Recognition of two-dimensional (2D) forms 3	Analysis of complex visual signs and sign groups (compositions) related to form and content. Analysis of relations between pictorial components in connection with content. Interpretation of visual emphases. Analysis of well-known visual signs in new context.
Visual recognition	Abstraction 1	Differentiation between and recognition of signs and sign groups; observe techniques of emphasis through methods of pictorial composition.
Visual interpretation	Abstraction 2	Interpretation of the relations between real-life images and visual signs. Interpretation of explanatory drawings, charts, and diagrams based on conventions and rules. Interpretation of relations among images, observation of changes (e.g., acceleration of movement).
Visual analysis	Abstraction 3	Employment of methods of form and function analysis: interpretation of diagrams, pictorial signs, groups of signs, and works of architecture and design.
Visual recognition	Symbolization 1	Recognition, definition, and naming of symbols, allegories, and visual metaphors in familiar contexts.
Visual interpretation	Symbolization 2	Separation, comparison, and analysis of symbols, allegories, and visual metaphors in partially familiar contexts.
Visual analysis	Symbolization 3	Analysis and assessment of symbols, allegories, visual signs, and metaphors in new contexts.
Visual recognition	Modality shift 1	Recognition of different experiences and modalities (seeing, hearing, smelling, touching, tasting) in images and signs; connection of familiar images and signs to different modalities.
Visual interpretation	Modality shift 2	Connection of different experiences, modalities (seeing, hearing, smelling, touching, tasting) to images. Connection of familiar and partially new signs with different modalities.
Visual analysis	Modality shift 3	Analysis of connections of sensory experiences in different modalities (seeing, hearing, smelling, touching, tasting) with visual signs.

**Table 3:** The system of tasks and visual patterns in the test according to the primary skill elements.

Subskills	Image Types	Number of Tasks	Number of Items
*Recognition and differentiation*: recognition of form and color, matching a part and the whole of the image, matching images represented through different media	Paintings and sculpture in classic historic styles, snapshots and phase photos, storybook illustrations	11	38
*Visual interpretation and recognition of connections:* recognizing depicted activities and objects; symbolization 1: attaching meaning to abstract images, symbolization 2: connecting messages of a musical sequence with abstract images, matching a literary work with its illustration	Maps, simple linear drawings, works of art, basic elements of visual language (dot, line, geometric shapes, patches), pictograms of everyday use, storybook illustrations	26	67
*Visual analysis:* reading graphs and charts, interpreting color symbols and spatial relations.	Instruction sheets, graphs and charts, photos, works of art	9	14

**Table 4:** Performance of boys and girls on paper and digital tests of the “produce” domain of visual communication.

Parameters	Paper-Based Test Scores		Digital Test Scores	
	Males (N = 57)	Females (N = 46)	Males (N = 151)	Females (N = 134)
Mean (%)	40.27	60.87	50.13	50.96
Std dev. (%)	16.99	16.81	20.10	18.73

(Paper-based test: F = 0.02; *p* = 0.90;*|t|* = −6.15; *p* < 0.001. Digital test: F = 0.60; *p* = 0.44; *|t|* = −0.36; *p* = 0.72).

**Table 5:** Digital test of visual communication: “produce” domain.

Category	Test
Number of items	23
Number of participants (N)	312
Cronbach’s alpha	0.82
Mean (%)	49.64
Std. Deviation (%)	20.62

**Table 6:** Distribution of test score by age in the “produce” domain of the visual communication test.

Age (Year)	N	Test Results		One-Way ANOVA			Significantly Different Groups Based on Dunnett’s T3 Test
		Mean (%)	Std Dev. (%)	F	*p*	η_p_^2^	
11	71	37.13	17.77	39.27	<0.001	0.30	{11:12} < {13:14}
12	74	44.60	16.76				
13	64	56.17	13.03				
14	77	64.24	17.38				

Note: Numbers in the comparison column between groups refer to subsamples by age group. The “<” sign indicates the direction of the significant difference.

**Table 7:** Gender-related results of the digital test of visual communication: “produce” domain.

Parameters	Students		Independent-Sample *t*-Test		
	Boys (N = 151)	Girls (N = 134)	Z	*p*	Cohen’s d
Mean (%)	50.13	50.96	−0.42	0.66	0.04
Std dev (%)	20.10	18.73			

Mann–Whitney test sig. *p* < 0.05; Classification scale of the effect size of Cohen’s d: small (d = 0.2), medium (d = 0.5), large (d = 0.8).

**Table 8:** Digital test of visual communication: “produce” domain. Results of the task-level analysis.

Category	Test Tasks (N = 312)						
	1. Color Contrast	2. Graphic Symbol 1.	3. Graphic Symb. 2.	4. Figure Comp. 1.	5. Figure Comp. 2.	6. Spatial Repr.	7. Visual Rhythm
Item number	2	6	5	3	2	4	2
Cronbach’s alpha	0.59	0.72	0.46	0.74	0.92	0.69	-
Mean (%)	41.46	58.65	48.08	52.72	52.55	47.13	53.69
Std. dev (%)	36.03	30.63	26.70	35.53	43.17	30.82	45.12

Color contrast: F = 8.22; *p* < 0.05; graphic symbol 1.: F = 4.04; *p* < 0.001; graphic symbol 2.: F = 41.44; *p* < 0.001; figurative composition 1.: F = 341.93; *p* < 0.001; figurative composition 2.: F = 0.80; *p* < 0.05; 2D spatial representation: F = 32.90; *p* < 0.001; visual rhythm: F = 19.24; *p* < 0.001.

**Table 9:** Digital test of visual communication: “produce” domain: scores of tablet and personal computer users.

Parameters	Device		Independent-Sample *t*-Test		
	Tablet (N = 182)	PC (N = 104)	Z	*p*	Cohen’s d
Mean (%)	49.66	52.29	−0.42	0.27	0.13
Std. dev (%)	18.83	20.54			

(Mann–Whitney test sig. *p* < 0.05). Classification scale of the effect size of Cohen’s d: small (d = 0.2), medium (d = 0.5), large (d = 0.8).

**Table 10:** Test reliability indicators by grade.

Grades	Number of Items	Cronbach’s α	N (Valid Tests)
Grade 4	57	0.87	416
Grade 5	64	0.89	217
Grade 6	72	0.89	482

**Table 11:** EAP/PV reliability indicators by Grade.

Grades	EAP/PV Reliability Indicators
Grade 4	0.84
Grade 5	0.83
Grade 6	0.85

**Table 12:** Results of the confirmatory factor analysis of the visual communication test by grade.

Model	χ^2^	*p*	df	CFI	TLI	RMSEA (95% CI)
Grade 4	1746.13	<0.001	703	0.679	0.658	0.063 (0.059–0.067)
Grade 5	3144.69	<0.001	1371	0.742	0.731	0.063 (0.060–0.066)
Grade 6	6701.85	<0.001	1431	0.587	0.569	0.057 (0.055–0.060)

Note: df = degrees of freedom; CFI = comparative fit index; TLI = Tucker–Lewis Index; RMSEA = root-mean-square error of approximation; χ^2^ and df are estimated by WLSMV.

**Table 13:** Visual communication skills’ test standard deviations and means by grades.

Grade	N	Minimum	Maximum	Mean (%)	Std. Dev (%)
4	432	0	55	67.57	15.47
5	338	0	63	67.11	14.77
6	486	0	69	67.56	15.02
Total	1256	0	69	67.44	15.10

## Data Availability

Not applicable.
